# Understanding the Impact of Social Stress on Serum Metabolome and Saliva Biomarkers in Growing–Finishing Pigs

**DOI:** 10.3390/ani15091228

**Published:** 2025-04-27

**Authors:** Marc Bagaria, Núria Tous, David Torrallardona, Jose Joaquín Cerón, Estefanía Pérez-Calvo, Wen Ren, Rosa Argamasilla, Emma Fàbrega

**Affiliations:** 1 Animal Welfare Program, Institute of Agrifood Research and Technology (IRTA), 17121 Monells, Spain; emma.fabrega@irta.cat; 2 Animal Nutrition Program, Institute of Agrifood Research and Technology (IRTA), 43120 Constantí, Spain; nuria.tous@irta.cat (N.T.); david.torrallardona@irta.cat (D.T.); 3 Interdisciplinary Laboratory of Clinical Analysis (Interlab-UMU), University of Murcia, 30100 Murcia, Spain; jjceron@um.es; 4 Animal Nutrition and Health, DSM-Firmenich, 4303 Kaiseraugst, Switzerland; estefania.perez-calvo@dsm-firmenich.com (E.P.-C.); rosa.argamasilla@dsm-firmenich.com (R.A.); 5 Animal Nutrition and Health, R&D Center, DSM-Firmenich, Bazhou 065700, China; wen.ren@dsm-firmenich.com

**Keywords:** stress, pig, blood metabolome, saliva biomarker

## Abstract

High levels of social stress are known to negatively impact pig welfare. The aim of this study was to evaluate the impact of social stress in growing–finishing pigs by measuring serum metabolome changes and saliva biomarkers. Seventy-two undocked pigs (thirty-six males and thirty-six females) were housed in single-sex pens of four, with the second dominant pig in each pen selected as the focal pig. A social challenge was conducted by mixing the focal pig with three new pigs in its home pen on two consecutive days on trial days 62–64. Saliva and blood samples were collected, and the pigs’ behaviour and body lesions were evaluated pre- and post-challenge. A total of 630 serum metabolites were analysed, 292 of which could be statistically compared using Biocrates WebIDQ v5 software. Salivary haptoglobin concentrations and the number of body lesions significantly increased after the challenge (*p* < 0.001), whereas the average daily weight gain decreased (*p* < 0.05). The serum showed decreases in essential amino acids (Thr, Met, and Phe), non-essential amino acids (Glu, Asn, Asp, Pro, and Tyr), betaine, ornithine, indoxyl sulphate, taurine, and some blood di- and triacylglycerols (q < 0.05), and increases in oleic, eicosanoic, eicosadienoic, and dihomo-gamma-linolenic acids; EPA; and DHA post-challenge (q < 0.05). Overall, the results suggest the potential of metabolomics as a tool providing a more holistic view of the impact of social stress.

## Simple Summary

Social stress is known to negatively impact the welfare, productivity, and health of pigs. However, finding proper biomarkers to evaluate the stress response is challenging because of the complexity of the response itself and potential confounding individual or methodological factors. The use of less invasive methods, like saliva sampling, or the development of more holistic tools that allow for the analysis of multiple metabolites with a single sample, may help explain the impact of stressful factors. In this study, a social challenge involving the mixing of pigs was conducted. Social stress increased the levels of haptoglobin in saliva; this acute-phase protein is released after inflammation and could be linked to the greater number of body lesions caused by infighting observed after the challenge. Out of 630 serum metabolites, 54 yielded significant differences after the social challenge. A decreased concentration of serum amino acids was observed, a result that could be related to the reduced feed intake and growth recorded. On the contrary, the concentration of lipid metabolites increased after the challenge; this rise was probably associated with the need to fulfill the energetic demands of the stress response. Overall, our results indicate that, despite the complexity of the data obtained and the lack of knowledge on the biological significance of some metabolites, metabolomics could have potential as a tool allowing us to more holistically understand the impact of stressful conditions like mixing. Thus, it can aid in the identification of social stress biomarkers.

## 1. Introduction

Pig husbandry systems can subject pigs to different types of stressors, such as social, metabolic, environmental, management, or immunologic stress [1]. While different definitions for stress have been proposed, a generally accepted approach from a physiological perspective defines stress as “the biological response elicited when an individual perceives a threat to its homeostasis” [2]. The dimensions of the stress response may vary according to the characteristics of the stressor—such as the nature, duration, frequency, intensity, circadian rhythmicity, controllability, and/or predictability of the stress—as well as individual traits such as gender, previous experiences, or age.

Among pigs, social stress is often caused by common management practices, such as cross-fostering during lactation, litter mixing at weaning, the use of mixed pens at the nursery and fattening stages, and transportation, among other practices that involve regrouping pigs with unfamiliar individuals [3]. Such events disrupt the established social structure within the group, leading to aggressive behaviours such as fighting, biting, intimidation, and competition for resources [4]. When pigs are exposed to these social-mixing events, they often exhibit various behavioural responses to the aggression they experience. These behavioural responses can be categorised as offensive, including head-to-head clashes, head knocks, biting, chasing, and postures or grunts intended to intimidate [5], or defensive, including freezing or attempting to escape confrontation [6]. While some level of aggression is natural during social-mixing events, prolonged or intense aggression can significantly impact the welfare of pigs, leading to physical injuries, reduced growth, and overall compromised well-being. Thus, social stress has been considered a relevant source of stress among pigs, and proper indicators of the magnitude of the impact have been studied.

Although there is no “golden standard” indicator with which to measure stress, in most methodologies, the aim has been to assess the two major components of this biological response: behaviour and physiology. The difficulties in defining these “golden standards” may be associated with the complexity of the stress response itself (i.e., the different pathways involved), the individual variation in some of the indicators, and/or the sampling methods’ potential interference with the response. For these reasons, combining different physiological indicators using an omics approach or combining different parameters like behaviour, physiology, and health may be more effective for evaluating the stress response. Moreover, this combined behavioural and physiological response depends, to a certain extent, on how the stressor is perceived, but some overarching common patterns have been described. According to traditional approaches like the General Adaptation System proposed by Selye [7], the stress response can be described in three stages. First, there is an alarm stage, during which an organism attempts to “fight or flee”. This phase of the stress response is controlled by the autonomous nervous sympathetic–adrenal–medullary system (SAM) through the secretion of catecholamines (i.e., dopamine, adrenaline, and noradrenaline) and behavioural changes mostly associated with activity. The second stage is resistance, in which the organism tries to adapt to the challenging conditions it is experiencing. This response involves the hypothalamic–pituitary–adrenocortical (HPA) axis influencing the release of glucocorticoids, feeding reduction, reproductive behaviour, and immune function, as well as the secretion of pancreatic hormones and protein/lipid balance. Lastly, there is the exhaustion stage, wherein the body’s resources are depleted and it is unable to maintain normal functioning, leading to behaviours like stereotypies or inactivity.

From a methodological point of view, trying to identify the best indicators with which to measure stress depends on a combination of feasibility and accuracy with respect to the stressor in question. For a social stressor, the behaviour indicators expected would be, as described before, an increase in agonistic interactions, resulting in an increase in the number of body lesions. In fact, in a recently published study, the authors used regrouping as an experimental model to evaluate the impact of social stress on behaviour and stress response and found a significant increase in body lesion quantities and changes in haematological parameters in regrouped pigs, and they even reported a potential emotional contagion spreading to non-regrouped pigs [8]. Social stress has also been found to increase susceptibility to gastrointestinal disease [9], alter immune cell recruitment and proliferation [4,10], and increase cortisol levels [11], acute-phase protein concentrations [12], and other haematological parameters [13]. Along these lines, the physiological indicators expected to vary in the face of a social stressor are those associated with changes in the resulting metabolites from the HPA and SAM systems.

Evaluating some of these physiological and behavioural indicators may pose a methodological challenge. Thus, measuring the catecholamines from the SAM system after a stress response is challenging [14], constituting one of the reasons why glucocorticoids have become one of the most widely measured stress indicators in many studies [1,2]. Despite being widely used, glucocorticoids present high individual variability and circadian rhythmicity and need to be interpreted with caution [1]. Developments in the omics field may help us overcome some of these limitations. In this vein, metabolomics could aid in the identification of biomarkers because it enables the identification of thousands of metabolites. Kasper et al. [15] suggested that omics could help researchers decipher the contributions of different physiological systems and identify candidate molecules that are representative of different physiological pathways, thus providing a more holistic view of the stress response. However, when Kasper et al. [15] reviewed the use of different omics methods in the evaluation of stress resilience and damaging behaviours, such as tail biting among pigs, they concluded that there are still important issues to be elucidated. For example, they identified a need to better understand the biology of the stress response and the molecular pathways involved in different growth stages and environments. More recently, the potential of metabolomics as a tool for evaluating pigs’ responses to stressful situations has been explored [16]. Several recent studies on metabolomics have identified potential biomarkers in different biofluids, such as blood [17,18] and saliva [19], in heat-stress situations. Post-weaning serum metabolome analysis has also revealed differences in piglets under different simulated pre-weaning stress conditions [20]. However, to the best of our knowledge, these tools have not yet been used for the identification of acute social stress biomarkers.

Importantly, instead of blood collection, the sampling of saliva, in addition to other types of samples like urine or faeces, hair, or feathers, is being used as a less invasive method. In saliva, among other compounds, cortisol can be used as a marker of stress, and haptoglobin can be employed as a marker of inflammation [21].

The objective of this study was to evaluate the use of blood metabolomics and saliva biomarkers for the assessment of the impact of social stress on growing–finishing pigs. For this purpose, regrouping was chosen as the stressor since it is a common practice on commercial farms. Our hypothesis was that the mixing events would disrupt the social order in the group, leading to aggressive behaviours and a stress response, in turn impacting blood metabolomics and saliva biomarkers and increasing the quantity of body lesions. The data presented in this paper are part of a broader study in which other indicators of the stress response (behaviour, microbiota, performance, and age) will be discussed.

## 2. Materials and Methods

### 2.1. Animals and Husbandry

This study was conducted at IRTA-Nutrition’s experimental farm at Centre Mas Bové in Constantí, Catalonia, Spain, from May to September 2023. The farm employed a closed-herd system with facilities for sows, weaner piglets, and growing–finishing pigs.

Thirty-six male pigs and thirty-six female pigs ([*Large White × Landrace*] *× Pietrain*) of approximately 51 days of age and weighing approximately 19.00 (SD 2.24) Kg with undocked tails from one batch of sows in IRTA’s sow herd were used. The pigs were housed in one room of the grower–finisher unit of the experimental farm, which consisted of 18 pens measuring 4.42 m^2^, where four pigs were allocated per pen. All pens were provided with one hopper feeder with four eating spaces and a drinker connected to a line that delivered an ad libitum automatic supply of potable drinking water. Food was provided to the pigs ad libitum and controlled daily, thus allowing continuous access to feed while avoiding spillage. All pens were provided with enrichment material that consisted of a chewable plastic bar hanging from a metal chain (MS Pig play material vertical, MS Schippers, Hapert, The Netherlands).

The room was outfitted with artificial lights, automatic heating, and forced ventilation via depression. The climate was controlled automatically by regulating the ventilation rate and heating (i.e., when the temperature in the room was above the specified temperature, the ventilation rate was automatically increased to reduce the temperature by introducing fresh air). Farm personnel also made manual adjustments to the ventilation system to ensure proper air quality. The temperature program was adjusted according to the standard program used at the farm for summer months, with a gradual decrease from 21 °C to 20 °C during the first month of the grower phase and from 20 °C to 18 °C during the last two months of the finisher phase. The room was lit via natural light through windows and tubular fluorescent lights. The artificial lights were programmed to provide 16 h of light and 8 h of darkness (the lights were switched on between 7:00 a.m. and 23:00 p.m.).

Three different dietary specifications were used according to phase (Table S1): the Grower-1 diet, supplied from trial days 0 to 36; the Grower-2 diet, supplied from trial days 36 to 71; and the Finisher diet, supplied from trial days 71 to 106. The diets were formulated such that they would meet or exceed the minimum nutrient requirements recommended by FEDNA [22], and they were provided in pellet form.

### 2.2. Design of the Experiment

**Figure 1:**
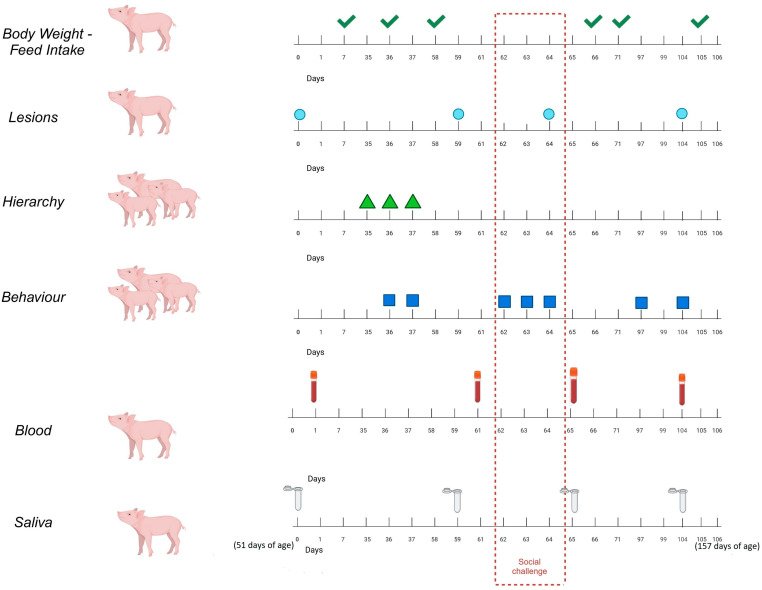
Summary of the different parameters measured on each day of the experiment arranged by period: baseline (day 0–7); pre-challenge (days 58–61); challenge (days 62–64); post-challenge (days 65–66); and pre-slaughter (days 97–106). On day 0, the pigs were 51 days old, and their average weight was 19.00 Kg; on day 106 of the trial (at 157 days old), they weighed 102.55 Kg.

Figure 1 summarises the different parameters measured on each day of the experiment. From day 0 to day 7, the parameters for the baseline period were assessed. On day 0, while the pigs were still in their original pens in the weaner facilities, saliva samples were taken from all the pigs in this study (N = 72), and their lesions were assessed. On day 1, blood samples were also taken from all the pigs in the study. The animals entered the fattening room on day 7, and they were distributed into the 18 pens according to blocks of sex, body weight, and pen location.

On days 35, 36, and 37, the hierarchy of the animals was assessed. A position in the hierarchy was allocated for each animal, and the second most dominant pig per pen was selected to be the “focal” animal, from which we took saliva and blood samples on the three following sampling days (n = 18). We followed the method used by da Fonseca de Oliveira et al. [23] to establish the hierarchy of the pigs within each pen. Live observations were made on three consecutive days between 10:00 and 14:00 h by two trained observers using the continuous sampling method [24]. Inter-observer reliability for hierarchy observations was calculated using a Kappa coefficient test, yielding a result of 0.89, indicating almost perfect agreement [24]. Before any observations were made, each animal was marked with a colour code for individual identification. The pens were divided between the two observers, and each pen was observed for 5 min. Then, the observers changed pens so that each observer could observe each of the 18 pens for the same amount of time. At the end of each observation day, each pen was observed for 30 min, amounting to a total observation time of 90 min per pen over the 3 days. Observations per pen were conducted at 5 min intervals and not continuously for 30 min/day to distribute them over different times of day according to the methodology reported by da Fonseca de Oliveira et al. [23] and Martin and Bateson [24]. During the observations, each agonistic interaction seen in a pen was noted, considering an agonistic interaction “an aggressive behaviour delivered by one pig towards another that lasted for more than 1 s”. For each agonistic interaction, the winner (determined at the point when the other individual involved in the fight showed a flight response and/or submissive behaviour) and the loser (the individual exhibiting escape movement during the event) were noted [23,25]. Using the data obtained, the dominance index (DI) was calculated for each individual in a pen according to the approach reported by Bowen and Brooks [26] using the following formula: DI = (wins − defeats)/(wins + defeats). The “focal” pigs (i.e., the second most dominant pig from each pen) were always kept in their original pens during the social stress challenge (involving the mixing of animals). We decided not to sample a random animal per pen but to establish a criterion for selecting a similar pig for each pen. Since we carried out a social challenge, we decided to avoid placing extreme pigs in the hierarchy and select the second most dominant one, according to an established methodology, for all the pens [23]. On day 36, the pigs were individually weighed.

From days 58 to 61, the parameters from the pre-challenge period were assessed. On day 58, all the pigs were individually weighed. On day 59, saliva samples were taken from the 18 focal animals, and all the animals’ lesions were assessed. On day 61, blood samples were taken from the 18 focal animals.

On days 62, 63, and 64, the challenge period, which consisted of instilling social stress by mixing the pigs, was initiated. For this purpose, the focal animal from each pen (the second most dominant pig in each pen, as ranked via behaviour evaluation) was always kept in its original pen. At 8:00 a.m. on day 62, two animals from each pen were removed and replaced by another two pigs (unknown to the focal pig) from another two pens that were of the same sex and the closest body weight for 24 h. At 8:00 a.m. on day 63, the three non-focal pigs from each pen were removed and replaced by one of the pigs that had been removed on day 62 and another two pigs (unknown to the focal pig) from another two pens that were of the same sex and closest body weight for an additional 48 h. The challenge ended at 8:00 on day 65, and all the animals were returned to their original pens. During the challenge days, the behaviours of all the pigs in the pens were assessed.

Lesions were evaluated at the end of day 64, which was considered the post-challenge evaluation of lesions. For consistency, only one observer was involved in lesion scoring. On days 65 and 66, the other parameters of the post-challenge period were assessed. On day 65, saliva and blood samples were taken from the 18 focal animals. On days 66 and 71, all the pigs were individually weighed. All saliva and blood samples were taken between 8:00 and 9:00 a.m. and 1 min before the start of the handling procedure to avoid that management itself could influence the results.

From days 97 to 106, the parameters of the pre-slaughter period were assessed. On day 104, the lesions of all the animals were assessed. On day 104, saliva samples were taken from the 18 focal animals; on day 105, all the pigs were individually weighed; and on day 106, blood samples were extracted from the 18 focal animals.

### 2.3. The Parameters Measured

#### 2.3.1. Performance

The pigs were individually weighed, and the feed intake per pen was controlled at the start and end of each grower phase and experimental period (i.e., days 7, 36, 58, 66, 71, and 106 of the trial). Body weight and feed intake were also controlled before and after the social challenge (i.e., days 58 and 66 of the trial). Average daily weight gain (ADG), average daily feed intake (ADFI), and the feed conversion ratio (FCR) were calculated for each phase: Grower-1 (days 7–36, age 58–87); Grower-2 before the challenge (days 36–58; age 87–109); Grower-2 during the challenge (days 58–66; age 109–117); Grower-2 after the challenge (days 66–71; age 117–122); Finisher (days 71–106; age 122–157).

#### 2.3.2. Lesions

The animals’ lesions were assessed at different times: baseline (day 0), pre-challenge (day 59), post-challenge (day 64 after the social challenge), and pre-slaughter (day 104). The lesions were always assessed after all the other parameters to avoid interactions with the results. In the assessments, two experienced observers entered the pen without touching or manipulating the pigs. For assessing the body lesions, we used an adapted version of the Welfare Quality protocol [27], where only one side of the animal was randomly selected (right or left), and their body lesions were evaluated and scored as follows: 0 (fewer than 15 scratches, open wounds, or wounds with blood); 1 (15 or more scratches with less than one/only one open wound or wound with blood); 2 (15 or more scratches with more than one open wound or wounds with blood). For ear lesions, an adapted version of the protocol developed by Diana et al. [28] was used, and both ears were evaluated and scored as follows: 0 (no ear lesions); 1 (scratches, red areas, superficial bites, or open wounds); and 2 (part of the ear tissue is missing, fresh blood is present, or there is evident necrosis). For tail lesions, an adapted version of the protocol developed by Lahrmann et al. [29] was used, and two different parameters were assessed. Tail damage severity was scored as follows: 0 (no visible tail lesions, or an earlier lesion has healed); 1 (minor superficial scratches); 2 (visible wounds and tissue damage); and 3 (the outer part of the tail has almost been bitten off, and the tip of the tail fell off during healing). Tail wound freshness was scored as follows: 0 (intact scabs, with the wounds covered by hard, dry scabs); 1 (non-intact scabs—wounds are covered with scabs, but cracks in the scabs and dried blood or fresh tissue are visible); 2 (fresh wounds with the skin broken, no scabs, and no blood—only weeping); and 3 (fresh wounds with a fresh lesion and fresh blood are visible).

#### 2.3.3. Behaviour

The behaviour of the animals was assessed at three times: pre-challenge (days 36 and 37), challenge (days 62, 63, and 64), and post-challenge (days 97 and 104). The behaviour of the animals was assessed using continuous sampling methods [24], which consisted of observing each pen for 5 min and recording every behaviour exhibited by any of the animals in the pen. The behaviours observed included affiliative, agonistic, enrichment exploration, tail-biting, and ear-biting behaviours (Table 1). In each period, each pen was observed 12 times for 5 min, leading to a total observation time of 60 min per pen, and each behaviour was expressed as the mean proportion of the behaviour per minute of observation. The observations were made between 9:00 and 15:00 h by three trained observers at the same time, alternating the pens observed to balance the observations between pens. Inter-observer reliability for behavioural observations was calculated using a Kappa coefficient test, the result of which was 0.81, indicating almost perfect agreement [24].

#### 2.3.4. Saliva Biomarkers

Saliva samples were obtained from all the pigs (N = 72) on day 0 (baseline) and only from the 18 focal pigs (one pig per pen) on days 59 (pre-challenge), 65 (post-challenge), and 104 (pre-slaughter). Only the saliva samples of the 18 focal pigs (with 1 pig per pen) at the different phases were analysed to determine their cortisol and haptoglobin levels (HPT). Saliva samples were always taken before any other parameters were assessed to avoid affecting the results via disturbance or handling. On the sampling days, saliva samples were taken by two experienced technicians in the home pens of the animals. The pigs were first offered a cotton sponge attached to forceps to chew on, and if, after 30 s, the animals did not voluntarily chew on it, the sponge was gently introduced into their mouths to stimulate voluntary chewing for a maximum of 3 min. Restraint was not necessary for any of the pigs. Once the sponge was soaked, it was put into a centrifugation tube. Once all samples were collected, centrifugation tubes were centrifuged for 10 min at 3000 rpm and transferred into Eppendorf tubes using a micropipette. The Eppendorf tubes were stored at −20 degrees until salivary analysis was conducted.

Cortisol was measured via an indirect competitive AlphaLisa assay (PerkinElmer Inc., Hopkinton, MA, USA) using a monoclonal antibody previously validated in pig saliva [30]. Haptoglobin was measured via a direct competitive AlphaLisa assay using a monoclonal antibody, which was also validated in pig saliva [31].

#### 2.3.5. Blood Metabolomics

For blood sampling, feed was removed overnight so that the pigs would be in a fasting state at the time of sampling (08:00 a.m.). The pigs were fasted overnight to ensure the animals were under homogeneous conditions at the time of sampling, as the conditions under which pigs are kept could have an impact on, for example, amino acid metabolites. Blood samples were taken from all the pigs (N = 72) on day 1 (baseline) and only from the 18 focal pigs (with one pig per pen) on days 61 (pre-challenge), 65 (post-challenge), and 106 (pre-slaughter). Only the blood samples of the 18 focal pigs were analysed for their serum metabolomes, including with respect to cortisol evaluation. The pigs were gently restrained with a snout rope by trained experimenters, and blood samples (ca. 4.5 mL) were obtained via vena cava cranialis venepuncture less than 1 min after restraint. Blood samples were collected in Vacutainer tubes with a clot activator and kept at an ambient temperature until they were centrifuged for 10 min at 3000 rpm for serum separation. Aliquots of serum were obtained and quickly stored at −80 °C (with a maximum of 30 min from centrifugation to storage) until analysis.

To identify alterations in metabolite profiles, we determined the serum metabolomes using a targeted metabolomics approach via the Biocrates MxP^®^ Quant 500 kit (Biocrates Life Sciences AG, Innsbruck, Austria), which detects up to 630 metabolites from 26 compound classes and cortisol in blood. The samples were processed according to the manufacturer’s instructions. The sample extracts, reference standards, and quality control procedures were analysed using ultra-high performance liquid chromatography (UHPLC) and flow injection analysis (FIA), both coupled with tandem mass spectrometry. An Agilent 1290 series UHPLC system coupled to a 6500  +  QTrap mass spectrometer equipped with an Ion-Drive Turbo V^®^ ESI source (both Sciex, Foster City, CA, USA) was used for the analysis. Chromatographic and mass-spectrometric parameters were set as indicated by the manufacturer of the kit. Data analysis was carried out using Biocrates WebIDQ v5 software. Blood cortisol levels were measured using the method used to analyse the metabolomes.

### 2.4. Data Processing and Statistical Analysis

The statistical analysis of performance, lesions, behaviour, and saliva parameters was conducted using RStudio version 4.3.2 [32]. The tests were two-sided, with an alpha value of 0.05. For the analysis of these studied parameters, different LMMs and GLMMs were used. These models included performance (i.e., weight gain, feed intake, and feed-to-gain ratio), lesions (i.e., body lesions, ear lesions, tail damage severity, and tail wound freshness), behaviour (the mean proportion of each behaviour per minute per pen), and saliva biomarkers (cortisol and haptoglobin) as response variables; gender (females and males) and period (pre-challenge, challenge (for performance and behaviour), and post-challenge) as fixed effects, including their interactions; and pen and individual pig ID as random factors, accounting for the fact that the measurements were repeated over time. Before running the models, normality and homoscedasticity were checked, and either LMM or GLMM was used depending on the outcome. Individual pigs were used as an experimental unit for performance, physiological, and lesion data, whereas a pen was the experimental unit for behavioural data. For the performance and behaviour data, post-hoc pairwise analysis was conducted for the results with significant outcomes. Differences were considered significant if *p* < 0.05, and if *p* < 0.1, a tendency was considered.

For the serum metabolomics analysis, chromatographic peak integration, metabolite quantification, and pool normalisation were conducted using Biocrates WebIDQ v5 software. Analyte concentrations below the limit of detection (LOD) were substituted with NA values, metabolites with a relative standard deviation (RSD) greater than 15% in the pool sample were filtered out, and concentrations were expressed in ng/mL. The data were subsequently exported in CSV format for statistical analysis. Statistical analysis was performed using Python version 3.10 with relevant statistical packages installed (pandas, statsmodels, numpy, and scipy). For each group comparison, features with more than 50% missing values were excluded from the analysis. The remaining missing values were imputed with one-fifth of the LOD value for each variable. The samples were then log-10-transformed, and *t*-tests were conducted with a *p*-value threshold of 0.05, adjusted to a false discovery rate (FDR) of 0.05. The results are given as q-values. For the other parameters, no data were excluded.

## 3. Results

### 3.1. Performance

Table S2 summarises the descriptive performance results, including initial and final body weight, weight gain, feed intake, and feed-to-gain ratio by gender and for each of the phases: Grower-1, Grower-2 before the challenge, Grower-2 during the challenge, Grower-2 after the challenge, and Finisher. Table 2 summarises the performance parameters during the Grower-2-before-the-challenge, Grower-2-during-the-challenge, and Grower-2-after-the-challenge periods, and the statistical results.

Regarding weight gain, significant effects were found for gender x period interaction (F2, 54 = 11.18, *p* < 0.0001), gender (F1, 54 = 6.22, *p* = 0.01), and period (F2, 54 = 23.94, *p* < 0.0001). Both males and females gained more weight in the pre-challenge period than in the challenge period, and in the post-challenge period, males returned to their pre-challenge weights, while the females’ weights remained at the challenge values. Regarding feed intake, only period showed a significant effect (F2, 36 = 37.84, *p* < 0.0001), with feed intake being higher in the pre-challenge period than in both the challenge and post-challenge periods and higher in the challenge period than in the post-challenge period. Regarding the feed-to-gain ratio, a significant gender x × period interaction (F2, 54 = 10.66, *p* = 0.0001) and a period effect were found (F2, 54 = 17.36, *p* < 0.0001), and there was also a tendency for gender differences (F1, 54 = 3.58, *p* = 0.06). Both males and females presented higher feed-to-gain ratios in the pre-challenge period than in the challenge period, and in the post-challenge period, males returned to their pre-challenge weights, while the weights of the females remained at the challenge values.

### 3.2. Lesions

**Figure 2:**
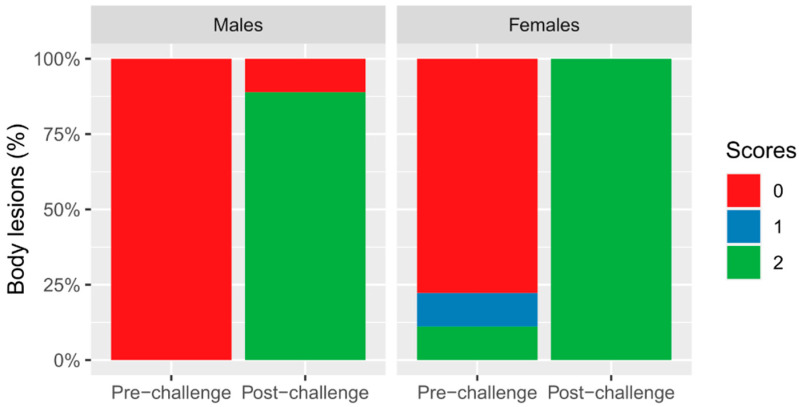
Stacked bar chart showing the different body lesion scores (0 = fewer than 15 scratches, open wounds, or wounds with blood; 1 = 15 or more scratches with fewer than one/only one open wound or a wound with blood; 2 = 15 or more scratches with more than one open wound or wound with blood). Pre-challenge: males (0: 100%), females (0: 77.77%; 1: 11.11%; 2: 11.11%). Post-challenge: males (0: 11.11%; 2: 88.88%), females (2: 100%).

Table S3 summarises the descriptive results regarding body lesions, ear lesions, tail damage severity, and tail wound freshness by gender, and for each period assessed: baseline, pre-challenge, post-challenge, and pre-slaughter. Table 3 summarises the percentage of pigs with a score of zero (no lesions) during the pre- and post-challenge periods and the corresponding statistical results, whereas Figure 2 presents the percentage of each score for body lesions.

Regarding body lesions, in both the pre-challenge and post-challenge periods, females presented more body lesions than males (X^2^ = 4.42, df = 1, *p* = 0.035), and there were more body lesions in the post-challenge period than in the pre-challenge period (X^2^ = 37.20, df = 1, *p* < 0.001). Regarding ear lesions, both males and females presented more ear lesions in the post-challenge period than in the pre-challenge period (X^2^ = 17.43, df = 1, *p* < 0.001). No significant differences were found regarding gender or period with respect to tail damage severity or tail wound freshness.

### 3.3. Behaviour

Table 4 summarises the results of the behaviour assessed by gender and for the three periods evaluated, i.e., pre-challenge, challenge, and post-challenge, and the corresponding statistical results.

Regarding affiliative behaviour, both males and females showed more affiliative behaviours in the challenge period than in the post-challenge period (X^2^ = 15.71, df = 2, *p* < 0.001). Regarding agonistic behaviour, both males and females showed more agonistic behaviour in the challenge period than in the pre-challenge and post-challenge periods (X^2^ = 35.97, df = 2, *p* < 0.001). Regarding enrichment exploration behaviour, both males and females showed more enrichment exploration in the post-challenge period than in the pre-challenge and challenge periods (X^2^ = 0.94, df = 1, *p* = 0.942). Regarding tail-biting behaviour, the results showed a significant period × gender interaction (X^2^ = 7.38, df = 2, *p* = 0.024) and gender differences (X^2^ = 7.89, df = 1, *p* = 0.004), with tail-biting behaviour being more common in the post-challenge period for females but less frequent for males. Regarding ear biting, no significant differences were found.

### 3.4. Saliva Biomarkers

Table S4 provides the descriptive results regarding saliva biomarkers arranged by gender and for each period assessed: baseline, pre-challenge, post-challenge, and pre-slaughter. Table 5 summarises the saliva biomarkers evaluated during the pre-challenge and post-challenge periods and the corresponding statistical results.

The results for saliva cortisol did not show significant gender or period differences. The results for saliva haptoglobin did show significant gender (F1, 32.26 = 7.17, *p* = 0.01) and period (F1, 19.97 = 114.83, *p* < 0.0001) differences, with both males and females presenting higher haptoglobin values in the post-challenge period than in the pre-challenge period and females presenting higher haptoglobin values in both periods.

### 3.5. Blood Metabolome

**Figure 3:**
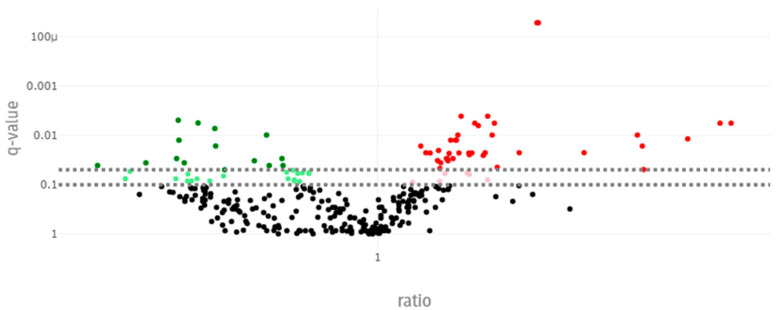
Volcano plot representing q values and ratios of metabolite concentrations during the pre-challenge and post-challenge periods. Green indicates a ratio below 1, red indicates a ratio above 1.

A quantitative analysis of a total of 630 metabolites was performed, and after a quality control check, a final total of 292 metabolites were used for further statistical analysis. Table S5 summarises the results regarding blood metabolomes by gender and for each period assessed: baseline, pre-challenge, post-challenge, and pre-slaughter. Table 6 summarises the results regarding blood metabolome during the pre-challenge and post-challenge periods and the corresponding statistical results. Given that no significant gender differences were found, gender was not included in the table. Only the fifty-four metabolites that showed q values (*p* values corrected for the false discovery rate) lower than 0.05, indicating significant differences between the pre-challenge and post-challenge periods, were included in Table 6. These metabolites are visually represented in Figure 3, in which they are colour-coded based on the ratio of their concentrations between different sample groups: green indicates a ratio below 1, signifying a decrease in concentration, while red indicates a ratio above 1, signifying an increase in concentration.

Regarding serum metabolomes, the most significant metabolites belong to the lipid and amino acid super pathways, as detailed in Table 6. Within these pathways, several specific changes were observed in the serum samples when comparing the pre-challenge and post-challenge periods. The statistical results showed that several amino acids (AAs), including Asn, Asp, Glu, Phe, Pro, Thr, and Tyr, significantly decreased in terms of serum concentrations in the post-challenge period when compared to the pre-challenge period. Additionally, the serum concentrations of amino acid derivatives such as Betaine, Orn, Ind-SO4, and Taurine were also significantly reduced. Conversely, the serum concentrations of the amino acid derivative creatinine, sphingomyelins, and long-chain fatty acids significantly increased in the post-challenge period. In contrast, hippuric acid levels decreased in the post-challenge period. Platelet levels significantly increased (q < 0.05) after the challenge, rising from 333 × 10^3^/mm^3^ to 436 × 10^3^/mm^3^.

Regarding plasma cortisol, the results did not show significant differences between the pre-challenge (26.10 ng/mL, SD 11.99 ng/mL) and post-challenge (33.85 ng/mL, SD 18.76 ng/mL) periods (q > 0.05).

## 4. Discussion

The main objective of this study was to evaluate the utility of blood metabolomics and saliva biomarkers in the evaluation of the impact of social stress among male and female pigs. This paper focuses on the results obtained after the implementation of a social challenge (mixing). The discussion is divided into two parts: the first part discusses the changes in saliva biomarkers, behaviour, and serum cortisol levels after the social challenge, and the second part discusses the metabolomics approach with a broader scope.

### 4.1. Impacts of the Social Challenge on Behaviour, Performance, Salivary Biomarkers, and Serum Cortisol

Regrouping pigs with unfamiliar conspecifics at different times is a common practice in commercial settings, including after weaning or for the fattening period. This practice is considered to jeopardise animal welfare since the social order is altered, and fights for the creation of new dominance hierarchies may break out, triggering social stress [3,4]. In our study, regrouping was used as a source of social stress to evaluate the impact on saliva and blood biomarkers.

Based on the behavioural observations and lesions, considered widely accepted measures of aggression and social conflict, the model chosen certainly created a social challenge. All the females and 97% of the males were scored as having severe body lesions after the challenge; these values can be compared to the proportions before the challenge, with 100% of males and 78% of females presenting no or few body lesions. This finding is in accordance with our hypothesis and agrees with findings previously reported in other studies [8,33,34]. Moreover, as expected, the social challenge increased agonistic interactions and worsened performance parameters. This effect was worse for females, who presented worse feed-to-gain ratios than males during the challenge phase. This result could be linked to the greater quantity of body lesions and higher haptoglobin levels in the females after the challenge, although it must be interpreted with caution, because females also presented more lesions before the challenge, and the comparison was made using a small number of animals. This is a somewhat unexpected result since most studies have reported that males are more aggressive and present more lesions (e.g., [35]). However, Camerlink et al. [36] reported instances of lethal gang aggression within stable groups of domestic pigs, occurring equally in female-only and mixed-sex groups, suggesting that females can display significant aggressive behaviour under specific conditions. Additionally, a study by Di Martino et al. [37] assessed the impact of sexual maturity on the welfare of heavy female pigs slaughtered at 36 weeks of age, and the findings indicated that sexually mature females exhibited a higher frequency of aggressive interactions and sustained more skin lesions compared to immune-castrated females. Further studies would be required to evaluate this somewhat unexpected result with regard to the effect of sex and how maturity or specific conditions could trigger aggressiveness in females. Other interesting aspects to be included in further studies include the differences between genetic lines and their potential interaction with sex with regard to aggressive behaviour.

However, when evaluating the impact of the social challenge on biomarkers, such clear-cut effects were not encountered. Neither salivary nor serum cortisol levels (included in the metabolomics array) significantly increased after the challenge. Several studies have reported increases in either salivary or plasma cortisol levels after mixing, a result associated with fights and agonistic encounters [11,38,39]. However, other studies, like the one recently published by Guevara et al. [8], also failed to observe an elevation in salivary or plasma cortisol levels in weanling piglets after repeated mixing. These authors collected saliva and blood 24 h after the final social-mixing procedure, once the pigs had returned to their original pens, and suggested that the post-social-mixing sampling point might not have reflected the peak cortisol concentration, as the cortisol concentration might have drop after the 24 h stress peak, which is usually expected to occur between 30 min and 3 h after the stressor [38]. In our study, we did not move the pigs from which saliva or blood samples were taken from their home pens (focal pigs), expecting to disentangle the effect of a novel pen from that of the social challenge. The focal pigs met their original partners at 8:00 a.m. after the 3 days of mixing, and samples were obtained within a time span of 2–4 h. This sampling schedule was chosen based on our hypothesis that even the act of rejoining with familiar pigs could trigger a certain period of social instability for the focal pigs; this was in turn based on previous results obtained using similar designs, implementing, for example, split marketing before slaughter delivery [40]. However, although our sampling was conducted closer to the point of social disruption than in the study by Guevara et al. [8], missing the peak of the higher cortisol response could be one of the reasons behind the results. Moreover, in our study, the mean values of serum cortisol, composed of free and protein-bound cortisol, and salivary cortisol, only composed of the free fraction, were higher after the social challenge, but these values were accompanied by high individual variability, which could also explain the lack of significance [1]. Accordingly, we cannot disregard the notion that a larger sample size could have yielded the expected results. Thus, although glucocorticoids are widely used as stress indicators, our results reinforce the need to be cautious because of some limitations related to the sampling technique used, age, circadian rhythmicity, gender, or other confounding factors [1].

In contrast, the haptoglobin concentrations in saliva were significantly higher after the social challenge, including in females. Haptoglobin is a member of the acute-phase proteins, which are blood proteins primarily synthesised in the liver and released into blood usually after inflammation, damage, and infection [1]. However, its link with stress is more inconsistent. Murata et al. [41] suggested that the release of catecholamines by the sympathetic autonomous system (SAM) induces the activity of proinflammatory cytokines like interleukin-6, which, in turn, induces hepatic acute-phase protein production. In our study, however, no biomarkers like chromogranin-A related to SAM activation were measured in order to clearly associate the haptoglobin increase with stress response. Moreover, cortisol levels did not significantly increase either. In contrast, tissular damage or inflammation could be present in the pigs associated with a clear increase in body lesions. Similarly, both body lesions and haptoglobin levels were more pronounced after the challenge, especially among females. Overall, these findings suggest that salivary haptoglobin is a suitable stress biomarker when a physical component is present, like in mixing, a finding in agreement with previous studies conducted using other factors like road transportation [42,43].

In this study, saliva was evaluated with regard to its accuracy as a less invasive alternative for blood sampling. It has been said that biomarkers of stress need to meet several criteria regarding validity, feasibility, and reliability. One of these is that the biomarker should be assessed from a sample that is easily obtainable and allows minimally invasive collection to guarantee there is a lower welfare impact on the individuals and reduce interference with the results [44]. In this study, saliva was certainly found to be easy to obtain; however, it failed to reveal significant differences in cortisol levels. A recent review by Cerón et al. [21] reported that although salivary cortisol has promising value as a less invasive marker, there are still issues that must be solved, like the high intraindividual variation, which likely explains the variability in the reference ranges reported in the literature. The coefficient of variation for saliva cortisol for the non-stressed pigs has been found to be 62% in some studies [45]. In this vein, other physiological indicators like acute-phase proteins—such as haptoglobin—or biomarkers of the adrenergic response, such as chromogranin-A or alpha-amylase, have been evaluated and/or combined with glucocorticoids to provide a better understanding of the stress response [1,46]. In the present study, haptoglobin levels in the saliva significantly increased and were in accordance with the increase in body lesion quantity. The increase in serum haptoglobin levels was less notable compared to the increase in the levels in saliva. Divergences between saliva and blood haptoglobin can occur in some situations since, for example, under conditions such as haemolysis or haemorrhage, serum haptoglobin levels can decrease [47] and mask increases due to inflammation. This would reinforce the need to tailor the physiological and behavioural indicators evaluated to the specific stressor and consider potential confounding effects. Moreover, exploring other non-invasive matrices like faeces or hair could provide further insights.

### 4.2. Impact of Social Challenge on Metabolomic Biomarkers

During the three days of social stress, the animals presented a reduction in feed intake of about 550 g/d and a consequent reduction in average daily gain of around 90% relative to pre-stress values. In line with these results, metabolome analysis also revealed a reduction in blood amino acid levels (some of which are essential for pigs, such as Thr, Met, and Phe, while others are non-essential, such as Glu, Asn, Asp, Pro, and Tyr), blood amino acid derivatives (such as betaine derived from glycine, ornithine derived from arginine, indoxyl sulphate derived from tryptophan, and taurine, which is a major component of bile, derived from methionine and cysteine), and some blood di- and triacylglycerols. The reductions in the concentrations of essential amino acids and di- and triacylglycerols could be a consequence of the reduced feed intake observed during this period. On the other hand, blood creatinine concentrations increased after the stress challenge. Creatinine is a product of the metabolism of arginine and proline (KEGG). This increase in creatinine concentration may be explained by increased muscle catabolism, serving as a potential mechanism by which these animals produce energy to overcome stress and/or compensate for the increased physical activity associated with fights and aggression between individuals. Interestingly, increases in creatinine levels have been described in humans after engaging in acute exercise [48]. Overall, the changes in the blood concentrations of amino acids and their derivatives could be explained by either the reduction in feed intake or increased amino acid catabolism. In addition, the utilisation of a specific AA for the synthesis of proteins for the immune system, such as haptoglobin, is provided either by dietary protein or the breakdown of skeletal muscle protein, which increases under certain circumstances. Consequently, the optimal AA profile required for growth and immune function may change during an acute or chronic state of immune system activation or a stress situation, as hypothesised by Kampman-van de Hoek [49]. In this study, we also observed a decrease in hippuric acid levels in the pigs’ serum after they were subjected to stressful stimuli. This decrease could be due to an increase in the excretion of this metabolite, which is produced in situations of stress and anxiety, via urine [50].

In a study assessing the effect of stress caused by transport and handling before slaughtering, pigs showed a cortisol response that was highly positively correlated with serum total n-3 fatty acids, suggesting a faster uptake of n-3 for biological functions such as stress control or energy supply in the brain [51], but we did not observe any such correlations of EPA or DHA with cortisol levels. In our study, significant increases in oleic, eicosanoic acid, eicosadienoic acid, dihomo-gamma-linolenic acid, EPA, and DHA were observed after the animals were subjected to stressful conditions. These are long-chain mono- and polyunsaturated fatty acids. Their increased concentrations in blood could be the consequence of low glucose levels caused by the reduced feed intake. In this case, glucose may be prioritised as an energy source for the brain as a part of the stress response, while the oxidation of fatty acids and amino acids may become the source for other tissues. In addition, dihomo-gamma-linolenic acid is a precursor to thromboxane B1, which acts as a vasoconstrictor, and it facilitates platelet aggregation [52]. This notion is in line with the changes in platelets observed after the stress challenge in our study, although all the values were within the physiological range for swine [53]. Previous reports have described a relation between platelet function and activation and stress [54], and platelets can also increase their numbers under stressful conditions [55]. The oxidation of the fatty acids EPA and DHA may result in the production of eicosanoids, which are anti-inflammatory and inflammation-resolving molecules and could modulate inflammatory status [56].

Stress increased all the levels of all the blood sphingomyelins evaluated in this study (most of them significantly). Increased sphingomyelin concentrations were also reported in males with severe post-traumatic stress disorder [57]. Diverse receptor types and responders to environmental stresses utilise the sphingomyelin pathway as a downstream effector system. The sphingomyelin pathway is a ubiquitous signalling system initiated by the hydrolysis of the plasma membrane phospholipid sphingomyelin to generate ceramide as a secondary messenger to induce apoptosis [58]. Like sphingomyelins, most of the hexocylceramides in our metabolome analysis significantly increased in number due to social stress. A recent differential metabolomics analysis study associated hexosylceramide HCER(26:1) with post-traumatic stress disorder (PTSD) in males [59]. In this study, an independent component analysis identified three metabolomic modules significantly associated with PTSD. These modules were significantly enriched in bile acid metabolism, fatty acid metabolism, and pregnenolone steroids, which are involved in innate immunity, inflammatory processes, and neuronal excitability, respectively. In addition, hexocylceramides are involved in the activation of inflammation [60], and therefore, their increased levels in our study could also be influenced by the inflammation produced in the pigs after subjection to the stressful conditions.

As previously described, cortisol is widely used as a stress indicator; however, it presents some limitations. Because of the complexity of the stress response, a more holistic tool such as metabolomics appears to be promising [61]. This metabolic study, including the analysis of a large cohort of metabolites, confirmed that lipid and amino acid metabolism are greatly influenced by stress. However, future research on the use of these potential metabolites as stress biomarkers requires further validation under different stressors. All in all, the current metabolomics study provides a wider approach to the metabolic pathways modified after a period of social stress.

## 5. Conclusions

Social factors, like mixing, create stressful conditions and thus impact pig behaviour and physiology, thereby impairing welfare and productivity. Finding proper biomarkers for stress has been challenging because of the complexity of the stress response itself. In this study, cortisol levels in saliva and blood were found to present greater individual variation and did not yield significant differences. In contrast, haptoglobin concentrations and body lesions significantly increased after the social challenge. This could be associated with the inflammatory response due to the lesions caused by the more frequent agonistic encounters observed during mixing. Moreover, 54 out of the 630 serum metabolites analysed using a metabolomics tool were found to significantly differ after the social challenge. Overall, while the levels of the metabolites associated with the amino acid pathways significantly decreased after the challenge, the levels of those associated with the lipid pathways increased. This could be associated with the decreased feed intake and growth during the challenge. Our results indicate the need to properly tailor the biomarkers chosen to the expected impact of each stressful factor and suggest that holistic tools like metabolomics may provide useful contributions after validation through further studies on the impact of social stressors like mixing. Our results also offer a broader understanding of the different metabolic pathways affected by social stressors.

## Figures and Tables

**Table 1:** Ethogram with a description of the behaviours evaluated via continuous sampling.

Behaviour	Description
Affiliative	An affiliative interaction, including sniffing, nosing, licking, or any social behaviour without a response from the receiving animal.
Agonistic	An aggressive interaction, including biting, knocking, or any social behaviour triggering a flight-or-fight response from the receiving animal.
Enrichment exploration	Playing or investigating by sniffing, nosing, licking, or chewing the enrichment material.
Tail biting	When one pig bites, chews, or pulls (using its mouth) the tail of another pig.
Ear biting	When one pig bites, chews, or pulls (using its mouth) the ear of another pig.

**Table 2:** Mean (and SD) of the performance parameters during the pre-challenge, challenge, and post-challenge periods according to gender.

Performance	Pre-Challenge		Challenge		Post-Challenge		*p*-Value		
	Males	Females	Males	Females	Males	Females	Gender	Period	Gender × Period
Weight gain (g/d)	874 (87.1) a	836 (91.5) a	449 (208) b	550 (126) b	964 (241) a	573 (204) b	0.01	<0.0001	<0.0001
Feed intake (g/d)	1846 (194) a	1884 (113) a	1584 (284) b	1674 (209) b	2098 (161) c	1988 (160) c	0.92	<0.0001	0.10
Feed-to-gain ratio	2.12 (0.16) a	2.27 (0.19) a	3.99 (1.23) b	3.17 (0.74) ab	2.32 (0.72) a	3.97 (1.78) b	0.06	<0.001	0.0001

a, b, and c within a row indicate significant differences between means.

**Table 3:** Percentage of lesions scored, showing the percentages of scores of zero (no lesions) during the pre-challenge and post-challenge periods according to gender.

Lesions	Pre-Challenge		Post-Challenge		*p*-Value		
	Males	Females	Males	Females	Gender	Period	Gender × Period
Body lesions (%)	100 a	77.77 b	11.11 c	0.00 d	0.03	<0.0001	0.99
Ear lesions (%)	66.66 a	55.55 a	0.00 b	22.22 b	0.33	<0.0001	0.10
Tail damage severity (%)	66.66	88.88	66.66	55.55	0.85	0.22	0.27
Tail wound freshness (%)	66.66	88.88	66.66	66.66	0.63	0.38	0.41

a, b, c, and d within a row indicate significant differences between percentages.

**Table 4:** Mean (and SD) of the behaviours assessed, showing the mean proportion of each behaviour per minute per pen during the pre-challenge, challenge, and post-challenge periods according to gender.

Behaviour	Pre-Challenge		Challenge		Post-Challenge		*p*-Value		
	Males	Females	Males	Females	Males	Females	Gender	Period	Gender × Period
Affiliative	0.255 (0.216) ab	0.246 (0.105) ab	0.307 (0.128) a	0.325 (0.100) a	0.183 (0.150) b	0.129 (0.119) b	0.77	<0.0001	0.85
Agonistic	0.118 (0.075) a	0.087 (0.045) a	0.244 (0.163) b	0.335 (0.202) b	0.083 (0.055) a	0.111 (0.065) a	0.41	<0.0001	0.24
Exploring enrichment	0.264 (0.174) a	0.231 (0.082) a	0.124 (0.040) b	0.111 (0.056) b	0.333 (0.163) c	0.388 (0.163) c	0.94	<0.0001	0.43
Tail biting	0.024 (0.022) a	0.031 (0.031) a	0.022 (0.018) a	0.029 (0.023) a	0.005 (0.016) b	0.061 (0.054) c	0.004	0.75	0.02
Ear biting	0.024 (0.042)	0.050 (0.044)	0.068 (0.052)	0.057 (0.039)	0.038 (0.048)	0.066 (0.066)	0.49	0.08	0.53

a, b, and c within a row indicate significant differences between incidences/minute.

**Table 5:** Means (and SDs) of the saliva biomarker parameters during the pre-challenge and post-challenge periods by gender.

Saliva Biomarkers	Pre-Challenge		Post-Challenge		*p*-Value		
	Males	Females	Males	Females	Gender	Period	Gender × Period
Cortisol (ng/mL)	148.05 (26.07)	152.18 (30.54)	156.88 (91.07)	172.62 (79.92)	0.50	0.78	0.54
Haptoglobin (ng/mL)	188.02 (138.23) a	271.43 (142.33) b	580.80 (421.95) c	1513.94 (1200.19) d	0.01	<0.0001	0.07

a, b, c, and d within a row indicate significant differences between means.

**Table 6:** Means (and SDs) of the blood metabolome parameters during the pre-challenge and post-challenge periods. Gender differences were not significant; thus, only the metabolites with significant period differences are provided (for a q-value lower than 0.05, ratios are provided).

Biochemical Name (+ Abbreviation)	Super and Sub Pathways	Pre-Challenge	Post-Challenge	q-Value	Ratio
	Amino Acid (Super)	(ng/mL)	(ng/mL)		
Asparagine (Asn)	Alanine and Aspartate Metabolism	9405 (4414)	5958 (2211)	0.0126	0.58
Aspartate (Asp)	Alanine and Aspartate Metabolism	5299 (1532)	4026 (876)	0.0099	0.73
Betaine	Glycine, Serine, and Threonine Metabolism	43,727 (10,842)	32,163 (8753)	0.0328	0.71
Creatinine	Creatine Metabolism	14,713 (2551)	16,441 (2571)	0.0165	1.13
Glutamate (Glu)	Glutamate Metabolism	42,993 (15,140)	33,176 (9637)	0.0408	0.74
Indoxyl sulphate (Ind-SO4)	Tryptophan Metabolism	281 (156)	402 (169)	0.0225	1.48
Methionine sulfoxide (Met-SO)	Methionine, Cysteine, SAM, and Taurine Metabolism	342 (187)	212 (97)	0.0295	0.57
Ornithine (Orn)	Urea Cycle and Arginine and Proline Metabolism	12,286 (4481)	8077 (2670)	0.0056	0.61
Phenylalanine (Phe)	Phenylalanine Metabolism	17,733 (6089)	14,259 (3296)	0.0297	0.77
Proline (Pro)	Urea Cycle and Arginine and Proline Metabolism	37,174 (14,935)	25,882 (7451)	0.0165	0.64
Taurine	Methionine, Cysteine, SAM, and Taurine Metabolism	22,987 (6419)	17,755 (3975)	0.0407	0.77
Threonine (Thr)	Glycine, Serine, and Threonine Metabolism	20,514 (7295)	12,620 (4698)	0.0050	0.58
Tyrosine (Tyr)	Tyrosine Metabolism	17,373 (6747)	11,885 (3379)	0.0073	0.64
	**Lipid (Super)**				
Cer(d18:1/18:0)	Ceramides	86 (43)	111 (32)	0.0255	1.34
Deoxycholic acid (DCA)	Secondary Bile Acid Metabolism	25 (20)	40 (31)	0.0492	2.09
Docosahexaenoic acid (DHA)	Long-Chain Polyunsaturated Fatty Acid (n3 and n6)	190 (177)	317 (222)	0.0225	1.77
Eicosapentaenoic acid (EPA)	Long-Chain Polyunsaturated Fatty Acid (n3 and n6)	34 (30)	52 (31)	0.0099	2.05
Octadecenoic acid (FA 18:1)	Long-Chain Monounsaturated Fatty Acid	23,323 (32,650)	49,785 (30,599)	0.0057	2.58
Eicosenoic acid (FA 20:1)	Long-Chain Monounsaturated Fatty Acid	968 (1356)	1291 (1006)	0.0057	2.66
Eicosadienoic acid (FA 20:2)	Long-Chain Polyunsaturated Fatty Acid (n3 and n6)	2545 (3980)	3130 (2320)	0.0118	2.36
Dihomo-gamma-linolenic acid (FA 20:3)	Long-Chain Polyunsaturated Fatty Acid (n3 and n6)	281 (495)	521 (423)	0.0165	2.08
Hex2Cer(d18:1/18:0)	Glycerolipid Metabolism	134 (57)	153 (53)	0.0232	1.22
Hex3Cer(d18:1/16:0)	Glycerolipid Metabolism	554 (92)	690 (122)	0.0041	1.26
Hex3Cer d18:1/24:2	Glycerolipid Metabolism	193 (43)	253 (59)	0.0057	1.31
HexCer(d18:1/16:0)	Glycerolipid Metabolism	238 (104)	281 (67)	0.0362	1.19
HexCer(d18:1/22:0)	Glycerolipid Metabolism	237 (55)	282 (56)	0.0295	1.21
HexCer(d18:1/23:0)	Glycerolipid Metabolism	178 (51)	211 (58)	0.0328	1.22
HexCer(d18:1/24:0)	Glycerolipid Metabolism	209 (54)	240 (58)	0.0202	1.18
HexCer(d18:1/24:1)	Glycerolipid Metabolism	517 (107)	635 (122)	0.0126	1.24
lysoPC a C20:40	Lysophospholipid	3472 (1136)	4143 (887)	0.0227	1.25
PC aa C38:4	Phosphatidylcholine (PC)	149,171 (60,900)	188,439 (43,783)	0.0228	1.30
PC aa C38:5	Phosphatidylcholine (PC)	22,747 (8738)	28,761 (5641)	0.0248	1.29
PC aa C38:6	Phosphatidylcholine (PC)	9782 (3376)	13,118 (4223)	0.0099	1.37
PC aa C40:6	Phosphatidylcholine (PC)	9395 (3341)	11,863 (3804)	0.0227	1.29
PC ae C36:4	Phosphatidylcholine (PC)	3181 (836)	3871 (463)	0.0126	1.22
PC ae C36:5	Phosphatidylcholine (PC)	1282 (323)	1723 (261)	0.0041	1.36
PC ae C38:5	Phosphatidylcholine (PC)	3978 (1035)	4925 (626)	0.0126	1.24
PC ae C38:6	Phosphatidylcholine (PC)	744 (171)	922 (141)	0.0099	1.25
PC ae C40:1	Phosphatidylcholine (PC)	539 (215)	714 (211)	0.0057	1.38
PC ae C40:6	Phosphatidylcholine (PC)	910 (220)	1084 (256)	0.0470	1.19
SM (OH) C16:1	Sphingomyelins	44,529 (8373)	50,599 (7288)	0.0225	1.14
SM (OH) C22:1	Sphingomyelins	3321 (587)	3822 (540)	0.0228	1.16
SM (OH) C24:1	Sphingomyelins	7101 (1582)	10,827 (2437)	0.0001	1.56
SM C16:0	Sphingomyelins	2202 (453)	3356 (712)	0.0001	1.55
SM C16:1	Sphingomyelins	94 (24)	125 (31)	0.0063	1.32
SM C18:1	Sphingomyelins	1634 (493)	2011 (396)	0.0295	1.23
SM C20:2	Sphingomyelins	816 (193)	996 (178)	0.0295	1.21
SM C22:3	Sphingomyelins	4269 (1165)	5017 (1029)	0.0326	1.18
SM C24:0	Sphingomyelins	5908 (1751)	7912 (1367)	0.0225	1.35
TG(17:0_36:3)	Glycerolipid Metabolism	1159 (763)	776 (461)	0.0499	0.65
TG(18:2_35:1)	Glycerolipid Metabolism	1445 (921)	878 (534)	0.0362	0.58
TG(18:2_36:4)	Glycerolipid Metabolism	13,184 (9547)	6489 (5316)	0.0410	0.46
TG(20:5_36:3)	Glycerolipid Metabolism	435 (508)	598 (219)	0.0445	1.39
	**Xenobiotics (Super)**				
Hippuric acid (HipAcid)	Benzoate Metabolism	5047 (2342)	2973 (1437)	0.0362	0.53

## Data Availability

All data from this study are provided in the manuscript and Supplementary Material. The raw data and the RStudio scripts used for the statistical analysis can be made available by the corresponding author upon reasonable request.

## Supplementary Materials

The following supporting information can be downloaded at https://www.mdpi.com/article/10.3390/ani15091228/s1. Table S1. Composition and calculated nutrient content of the diets during the three different phases, Table S2. Means (and SDs) of the performance parameters during the different periods and arranged by gender, Table S3. Percentages of the lesions scored, showing the percentage of scores of zero (no lesions), during the different periods and according to gender, Table S4. Means (and SDs) of the saliva biomarker parameters during the different periods and arranged by gender, Table S5. Means (and SDs) of the blood metabolome parameters during the different periods and according to gender.
