# Supplementary material for: Understanding the Impact of Social Stress on Serum Metabolome and Saliva Biomarkers in Growing–Finishing Pigs
Source: Animals (Basel). 2025 Apr 27;15(9):1228. doi: 10.3390/ani15091228 (PMC12071068; doi:10.3390/ani15091228)
Supplement: Supplementary file 1 [file animals-15-01228-s001.zip › animals-3540043-supplementary.pdf]

Supplementary material

Table S1. Composition and calculated nutrient content of the diets during the three different phases.

**Ingredients (%)**	**Grower-1**	**Grower-2**	**Finisher**
Corn	20.00	10.00	-
Wheat	25.00	30.00	38.17
Barley	26.94	34.79	39.32
Soybean meal (48% CP)	19.96	18.20	15.84
Sunflower meal (37% CP)	3.47	2.13	1.58
Animal fat	2.03	2.39	2.69
L-lysine HCl 98	0.19	0.16	0.15
L-threonine	0.04	0.03	0.02
DL- methionine	0.03	0.02	0.02
Calcium carbonate	0.25	0.32	0.38
Dicalcium phosphate	1.25	1.13	1.02
Salt	0.43	0.41	0.40
Vitamin and mineral premix[1]	0.40	0.40	0.40
Noxyfeed [2]	0.02	0.02	0.02
**Calculated nutrient content**			
ME, Kcal/kg	3180	3178	3175
Crude protein, %	18.00	17.50	17.00
Crude fibre, %	3.44	3.45	3.50
Fat, %	4.00	4.23	4.40
Ash, %	4.92	4.83	4.72
SID lysine, %	0.89	0.83	0.77
SID threonine, %	0.58	0.54	0.50
SID methionine, %	0.28	0.26	0.24
SID Met+Cys, %	0.55	0.53	0.51
SID tryptophan, %	0.19	0.18	0.18
Calcium, %	0.67	0.66	0.65
Total phosphorus, %	0.60	0.57	0.55
Digestible phosphorus, %	0.28	0.27	0.25

[1]Provided per kg feed: vitamin A (E 672) 5500 UI; vitamin D_3 (E 671) 1100 UI; vitamin E (alfa tocopherol) 25 mg; vitamin B_1 0.5 mg; vitamin B_2 1.4 mg; vitamin B_6 1 mg; vitamin B_{12} 8 µg; vitamin K_3 0.5 mg; calcium pantothenate 5.6 mg; nicotinic acid 8 mg; choline 120 mg; Fe (E 1) (from $FeSO_4{\cdot}7H_2O$) 80 mg; I (E 2) (from $Ca(I_2O_3)_2$) 0.5 mg; Co (E 3) (from $2CoCO_3{\cdot}3Co(OH)_2{\cdot}H_2O$) 0.4 mg; Cu (E 4) (from $CuSO_4{\cdot}5H_2O$) 5 mg; Cu (E 4) (from amino acids chelate) 5 mg; Mn (E 5) (from MnO) 40 mg; Zn (E 6) (from ZnO) 100 mg; Se (E 8) (from Na_2SeO_3) 0.25 mg.

[2]ITPSA, Barcelona, Spain. Contains BHT+ propyl gallate (56%) and citric acid (14%).

Table S2. Mean (and SD) of the performance parameters during the different periods and by gender.

Performance	Grower-1				Grower-2						Finisher	
	Baseline (0-7 d)		(7-36 d)		Pre-challenge (36-58 days)		Challenge (58-66 days)		Post-challenge (66-71 days)		(71-106 days)	
	Mal.	Fem.	Mal.	Fem.	Mal.	Fem.	Mal.	Fem.	Mal.	Fem.	Mal.	Fem.
Initial body weight (Kg)	19.2 (2.35)	18.7 (2.31)	24.3 (2.57)	24.1 (2.00)	46.4 (5.96)	47.2 (3.87)	65.6 (6.91)	65.6 (4.44)	69.0 (7.70)	70.0 (4.37)	74.0 (7.25)	72.9 (4.94)
Final body weight (Kg)	24.3 (2.57)	24.1 (2.00)	46.4 (5.96)	47.2 (3.87)	65.6 (6.91)	65.6 (4.44)	69.2 (7.70)	70.0 (4.37)	74.0 (7.25)	72.9 (4.94)	103.1 (8.13)	99.9 (5.70)
Weight gain (g/d)	641 (67.3)	664 (88.5)	761 (125)	799 (67.3)	874 (87.1)	836 (91.5)	449 (208)	550 (126)	964 (241)	573 (204)	857 (69.2)	796 (102)
Feed intake (g/d)	788 (79.4)	831 (25.1)	1459 (192)	1518 (109)	1846 (194)	1884 (113)	1584 (284)	1674 (209)	2098 (161)	1988 (160)	2291 (206)	2181 (175)
Feed to gain ratio	1.23 (0.10)	1.27 (0.18)	1.92 (0.10)	1.90 (0.06)	2.12 (0.16)	2.27 (0.19)	3.99 (1.23)	3.17 (0.74)	2.32 (0.72)	3.97 (1.78)	2.67 (0.14)	2.76 (0.23)

Mal.= Males; Fem.=Females

Table S3. Percentage of the lesions scored, showing the percentage of score 0 (no lesions), during the different periods and by gender.

Lesions	Baseline		Pre-challenge		Post-challenge		Pre-slaughter	
	Males	Females	Males	Females	Males	Females	Males	Females
Body lesions	77.77	100	100	77.77	11.11	0.00	88.88	100
Ear lesions	77.77	77.77	66.66	55.55	0.00	22.22	55.55	44.44
Tail severity	66.66	88.88	66.66	88.88	66.66	55.55	88.88	88.88
Tail freshness	66.66	88.88	66.66	88.88	66.66	66.66	88.88	88.88

Table S4. Mean (and SD) of the saliva biomarkers parameters, during the different periods and by gender.

Saliva biomarkers	Baseline		Pre-challenge		Post-challenge		Pre-slaughter	
	Males	Females	Males	Females	Males	Females	Males	Females
Cortisol (ng/mL)	202.58 (70.50)	171.90 (70.39)	148.05 (26.07)	152.18 (30.54)	156.88 (91.07)	172.62 (79.92)	167.10 (98.71)	181.25 (113.90)
Haptoglobin (ng/mL)	1422.63 (2025.42)	1796.64 (1233.36)	188.02 (138.23)	271.43 (142.33)	580.80 (421.95)	1513.94 (1200.19)	203.92 (112.77)	284.52 (106.22)

Table S5. Mean (and SD) of the blood metabolome parameters during the different periods and by gender.

Biochemical name (abbreviation)	Super and Sub Pathway	Baseline (ng/mL)		Pre-challenge (ng/mL)		Post-challenge (ng/mL)		Pre-slaughter (ng/mL)	
		Males	Females	Males	Females	Males	Females	Males	Females
	Amino acid (Super)								
Asparagine (Asn)	Alanine and Aspartate Metabolism	8869 (1550)	8881 (2592)	10203 (3721)	8608 (5111)	6852 (2756)	4953 (553)	4670 (6579)	1643 (6251)
Aspartate (Asp)	Alanine and Aspartate Metabolism	7914 (1060)	8383 (2520)	5400 (1410)	5198 (1726)	4279 (1056)	3742 (552)	4198 (3644)	597 (2890)
Betaine	Glycine, Serine and Threonine Metabolism	22897 (6720)	35639 (22628)	45745 (9593)	41709 (12190)	35606 (8489)	28290 (7770)	44185 (47581)	6167 (47990)
Creatinine	Creatine Metabolism	8282 (1288)	9184 (2592)	14652 (2429)	14773 (2815)	16713 (3103)	16134 (1973)	21458 (18531)	3139 (19222)
Glutamate (Glu)	Glutamate Metabolism	56595 (13936)	57144 (15896)	42825 (13518)	43162 (17444)	35436 (10881)	30634 (7938)	31072 (34842)	4622 (26393)
Indoxyl sulfate (Ind-SO4)	Tryptophan Metabolism	369 (183)	413 (296)	238 (95)	325 (196)	422 (157)	379 (190)	429 (510)	248 (581)
Methionine sulfoxide (Met-SO)	Methionine, Cysteine, SAM and Taurine Metabolism	162 (42)	180 (77)	364 (191)	321 (192)	238 (113)	183 (71)	178 (248)	102 (249)
Ornithine (Orn)	Urea cycle; Arginine and Proline Metabolism	7946 (1523)	8207 (2147)	12315 (4048)	12256 (5126)	8036 (3415)	8122 (1720)	7255 (9644)	2710 (10004)
Phenylalanine (Phe)	Phenylalanine Metabolism	11644 (2660)	12267 (3817)	18923 (5566)	16544 (6681)	14873 (3550)	13569 (3066)	11407 (16814)	2506 (13792)
Proline (Pro)	Urea cycle; Arginine and Proline Metabolism	26369 (3058)	29480 (8967)	38191 (13268)	36157 (17194)	28455 (9327)	22986 (3061)	23371 (31032)	6118 (30265)
Taurine	Methionine, Cysteine, SAM and Taurine Metabolism	19341 (5389)	19760 (6357)	23808 (7643)	22167 (5257)	17387 (2526)	18170 (5534)	20497 (21178)	5695 (17883)
Threonine (Thr)	Glycine, Serine and Threonine Metabolism	24498 (19831)	26185 (28880)	22417 (6262)	18611 (8107)	12092 (5011)	13213 (4581)	10099 (17277)	2439 (15557)
Tyrosine (Tyr)	Tyrosine Metabolism	8120 (2430)	7622 (1896)	18648 (6841)	16098 (6804)	11916 (4273)	11850 (2287)	10332 (16624)	3561 (15078)
	Lipid (Super)								
Cer(d18:1/18:0)	Ceramides	95 (36)	90 (18)	85 (56)	87 (26)	99 (28)	125 (32)	109 (105)	50 (106)
Deoxycholic acid (DCA)	Secondary Bile Acid Metabolism	22 (12)	14 (7)	26(21)	23 (20)	36 (19)	43 (41)	243 (49)	39 (60)

Docosahexaenoic acid (DHA)	Long Chain Polyunsaturated Fatty Acid (n3 and n6)	620 (322)	661 (281)	197 (245)	182 (80)	235 (116)	409 (282)	326 (183)	67 (173)
Eicosapentaenoic acid (EPA)	Long Chain Polyunsaturated Fatty Acid (n3 and n6)	80 (44)	51 (16)	38 (42)	30 (18)	45 (27)	60 (35)	61 (48)	17 (34)
Octadecenoic acid (FA 18:1)	Long Chain Monounsaturated Fatty Acid	50494 (29934)	29979 (8220)	24187 (39052)	22459 (27177)	43439 (29544)	56924 (32133)	62726 (40593)	31663 (33359)
Eicosenoic acid (FA 20:1)	Long Chain Monounsaturated Fatty Acid	1084 (773)	706 (236)	1447 (2012)	650 (760)	1142 (888)	1440 (1154)	1341 (975)	730 (981)
Eicosadienoic acid (FA 20:2)	Long Chain Polyunsaturated Fatty Acid (n3 and n6)	3121 (2580)	1773 (516)	5190 (6613)	1223 (1249)	2959 (2167)	3279 (2586)	2725 (2149)	1667 (2325)
Dihomo-gamma-linolenic acid (FA 20:3)	Long Chain Polyunsaturated Fatty Acid (n3 and n6)	667 (542)	376 (137)	348 (681)	214 (215)	450 (361)	600 (496)	432 (374)	276 (341)
Hex2Cer(d18:1/18:0)	Glycerolipid Metabolism	71 (17)	113 (17)	110 (38)	158 (65)	129 (34)	181 (58)	188 (179)	54 (195)
Hex3Cer(d18:1/16:0)	Glycerolipid Metabolism	526 (207)	573 (152)	546 (84)	561 (103)	672 (107)	709 (141)	630 (585)	97 (600)
Hex3Cer d18:1/24:2	Glycerolipid Metabolism	169 (53)	192 (67)	185 (44)	201 (44)	232 (49)	276 (64)	252 (209)	38 (190)
HexCer(d18:1/16:0)	Glycerolipid Metabolism	225 (63)	233 (58)	235 (134)	241 (70)	264 (48)	300 (82)	375 (310)	70 (269)
HexCer(d18:1/22:0)	Glycerolipid Metabolism	183 (51)	258 (63)	220 (40)	254 (64)	256 (52)	312 (48)	357 (300)	55 (288)
HexCer(d18:1/23:0)	Glycerolipid Metabolism	123 (35)	161 (35)	161 (49)	195 (50)	185 (41)	239 (63)	222 (212)	35 (231)
HexCer(d18:1/24:0)	Glycerolipid Metabolism	158 (40)	204 (41)	190 (43	229 (60)	221 (46)	262 (65)	275 (257)	33 (253)
HexCer(d18:1/24:1)	Glycerolipid Metabolism	432 (101)	515 (105)	491 (99)	543 (113)	620 (111)	653 (138)	833 (635)	88 (595)
lysoPC a C20:4	Lysophospholipid	4692 (725)	5315 (1275)	3493 (1026)	3451 (1300)	4158 (679)	4127 (1126)	3524 (4709)	1115 (4508)
PC aa C38:4	Phosphatidylcholine (PC)	171552 (45099)	198878 (47421)	149398 (77234)	148944 (43773)	185611 (43105)	191620 (47289)	190465 (169674)	37050 (169828)
PC aa C38:5	Phosphatidylcholine (PC)	28543 (6856)	31492 (6620)	23247 (11273)	22247 (5883)	27757 (4055)	29890 (7154)	33418 (29786)	4525 (28314)
PC aa C38:6	Phosphatidylcholine (PC)	17697 (5517)	22752 (6671)	9457 (4071)	10106 (2723)	12025 (2638)	14347 (5437)	11993 (9987)	1836 (10285)
PC aa 40:6	Phosphatidylcholine (PC)	17444 (6667)	23762 (7417)	8871 (4064)	9920 (2565)	11104 (3342)	12717 (4328)	10989 (9219)	2720 (9950)
PC ae C36:4	Phosphatidylcholine (PC)	3439 (1074)	4035 (1232)	3220 (1086)	3142 (550)	3812 (482)	3937 (464)	4093 (3733)	657 (3543)
PC ae C36:5	Phosphatidylcholine (PC)	1248 (291)	1583 (549)	1281 (422)	1282 (209)	1691 (233)	1759 (302)	1699 (1553)	320 (1516)

PC ae C38:5	Phosphatidylcholine (PC)	4649 (1395)	5549 (1706)	4097 (1331)	3858 (688)	4962 (666)	4883 (620)	4900 (4504)	884 (4106)
PC ae C38:6	Phosphatidylcholine (PC)	1022 (312)	1211 (353)	752 (228)	736 (101)	901 (136)	946 (153)	924 (811)	162 (766)
PC ae C40:1	Phosphatidylcholine (PC)	672 (220)	721 (158)	552 (264)	526 (168)	706 (207)	723 (229)	699 (734)	238 (583)
PC ae C40:6	Phosphatidylcholine (PC)	1394 (403)	1879 (657)	895 (252)	926 (196)	1067 (297)	1103 (220)	1002 (944)	141 (916)
SM (OH) C16:1	Sphingomyelins	43425 (9298)	48490 (12388)	44601 (8914)	44457 (8338)	50201 (7215)	51048 (7842)	53803 (48027)	7738 (46377)
SM (OH) C22:1	Sphingomyelins	2891 (432)	3288 (859)	3393 (596)	3249 (605)	3787 (447)	3861 (659)	3793 (3790)	541 (3586)
SM (OH) C24:1	Sphingomyelins	7551 (2291)	8341 (2665)	6846 (1559)	7355 (1656)	10553 (1600)	11136 (3231)	10050 (8961)	1825 (8727)
SM C16:0	Sphingomyelins	2039 (531)	2361 (754)	2153 (447)	2252 (481)	3284 (569)	3438 (880)	3219 (2736)	440 (2664)
SM C16:1	Sphingomyelins	93 (17)	96 (28)	90 (30)	98 (18)	123 (21)	126 (41)	145 (124)	21 (113)
SM C18:1	Sphingomyelins	1203 (272)	1511 (456)	1602 (545)	1665 (467)	2018 (413)	2002 (405)	1926 (2124)	466 (1944)
SM C20:2	Sphingomyelins	569 (116)	685 (236)	810 (210)	821 (186)	1009 (163)	982 (203)	887 (955)	203 (923)
SM C22:3	Sphingomyelins	4014 (1023)	4454 (1426)	4429 (1242)	4109 (1133)	5298 (1119)	4701 (880)	5029 (5506)	1052 (4364)
SM C24:0	Sphingomyelins	5829 (1493)	6536 (1817)	5709 (1757)	6107 (1827)	7918 (1068)	7905 (1723)	6871 (6464)	1409 (6171)
TG(17:0_36:3)	Glycerolipid Metabolism	1097 (510)	1145 (763)	1372 (949)	945 (484)	832 (592)	714 (276)	518 (1091)	717 (1098)
TG(18:2_35:1)	Glycerolipid Metabolism	1192 (558)	1288 (834)	1716 (1062)	1173 (712)	971 (677)	773 (325)	526 (1268)	789 (1272)
TG(18:2_36:4)	Glycerolipid Metabolism	14649 (6038)	10786 (4532)	15200 (9360)	11168 (9846)	6931 (6750)	5991 (3462)	3590 (5578)	4193 (7164)
TG(20:5_36:3)	Glycerolipid Metabolism	1177 (474)	836 (143)	533 (706)	337 (166)	571 (186)	628 (262)	537 (562)	295 (508)
	Xenobiotics (Super)								
Hippuric acid (HipAcid)	Benzoate Metabolism	3927 (874)	5078 (5479	5479 (2639)	4615 (2066)	3602 (1598)	2265 (848)	2841 (3821)	1990 (3448)
